# Detection and Characterization of a Novel Reassortant Mammalian Orthoreovirus in Bats in Europe

**DOI:** 10.3390/v7112908

**Published:** 2015-11-11

**Authors:** Davide Lelli, Ana Moreno, Andrej Steyer, Tina Naglič, Chiara Chiapponi, Alice Prosperi, Francesca Faccin, Enrica Sozzi, Antonio Lavazza

**Affiliations:** 1 Experimental Zooprophylactic Institute of Lombardy and Emilia-Romagna, Via Bianchi 9, 25124 Brescia, Italy; anamaria.morenomartin@izsler.it (A.M.); chiara.chiapponi@izsler.it (C.C.); alice.prosperi@izsler.it (A.P.); francesca.faccin@izsler.it (F.F.); enrica.sozzi@izsler.it (E.S.); antonio.lavazza@izsler.it (A.L.); 2 Institute of Microbiology and Immunology, Faculty of Medicine, University of Ljubljana, Zaloška 4, SI-1000 Ljubljana, Slovenia; Andrej.Steyer@mf.uni-lj.si (A.S.); Tina.Naglic@mf.uni-lj.si (T.N.)

**Keywords:** *Orthoreovirus*, bats, full-genome sequence, reassortment

## Abstract

A renewed interest in mammalian orthoreoviruses (MRVs) has emerged since new viruses related to bat MRV type 3, detected in Europe, were identified in humans and pigs with gastroenteritis. This study reports the isolation and characterization of a novel reassortant MRV from the lesser horseshoe bat (*Rhinolophus hipposideros*). The isolate, here designated BatMRV1-IT2011, was first identified by electron microscopy and confirmed using PCR and virus-neutralization tests. The full genome sequence was obtained by next-generation sequencing. Molecular and antigenic characterizations revealed that BatMRV1-IT2011 belonged to serotype 1, which had not previously been identified in bats. Phylogenetic and recombination detection program analyses suggested that BatMRV1-IT2011 was a reassortant strain containing an S1 genome segment similar to those of MRV T1/bovine/Maryland/Clone23/59 and C/bovine/Indiana/MRV00304/2014, while other segments were more similar to MRVs of different hosts, origins and serotypes. The presence of neutralizing antibodies against MRVs has also been investigated in animals (dogs, pigs, bovines and horses). Preliminary results suggested that MRVs are widespread in animals and that infections containing multiple serotypes, including MRVs of serotype 1 with an *S1* gene similar to BatMRV1-IT2011, are common. This paper extends the current knowledge of MRVs and stresses the importance to continue and improve MRV surveillance in bats and other mammals through the development and standardization of specific diagnostic tools.

## 1. Introduction

Orthoreoviruses, with mammalian orthoreovirus (MRV) as the type species, are non-enveloped viruses having a segmented dsRNA genome. MRVs have a wide geographic distribution, can infect virtually all mammals, including humans, and are responsible for symptomatic or asymptomatic infections [1]. Three MRV serotypes have been recognized based on the capacity of anti-MRV sera to neutralize viral infectivity and inhibit hemagglutination [2]. 

Members of the genus *Orthoreovirus* contain 10 genome segments, which are designed as large (L, three segments), medium (M, three segments) or small (S, four segments) based on their electrophoretic mobility [3].

Neutralization and hemagglutinin activities are restricted to a single reovirus gene segment, *S1* [4]. This encodes the σ1 protein, located on the outer capsid of the virion, that is responsible for viral attachment to cellular receptors and determines the reovirus serotype [5]. An analysis of the *S1* genes of MRVs has shown a strict correlation between sequence similarities and viral serotypes [3,6,7]. Conversely, the other genome segments show no correlation to viral serotype, suggesting that MRV reoviruses have evolved independently of serotypes [8].

A renewed interest in *Orthoreovirus* has occurred for several reasons, mainly, but not only, related to public health concerns. In the last few years, MRVs have often been determined to be responsible for severe human illnesses, including hemorrhagic enteritis, acute respiratory infections and encephalitis [9,10,11,12,13,14,15]. The zoonotic potential of reoviruses has already been described and discussed elsewhere [10,11]. Novel MRVs have recently been identified in several hosts, including bats in Italy and Germany [16,17], and a novel *Orthoreovirus*, with a high similarity to the MRVs found in bats in Europe, was detected in Slovenia from a child requiring hospitalization due to acute gastroenteritis [15]. Recently, a virulent MRV containing an *S1* segment that was strongly homologous to bat MRV3 was detected from diarrheic pigs in the United States [18]. In addition, a bat-borne fusogenic *Orthoreovirus* with zoonotic potential was detected from healthy flying foxes (*Pteropus vampyrus*) legally imported from Indonesia to Europe [19]. Moreover, the segmented nature of *Orthoreovirus* genomes poses risks for the potential formation of novel reassortant viruses with unpredictable biological properties. Finally, the scientific community’s awareness of the importance of reovirological studies has increased because MRVs are being evaluated as oncolytic agents in experimental cancer therapies [20].

Bats, as the most abundant, assorted and geographically-disperse vertebrates, are increasingly known as reservoirs of viruses that can cross species barriers to infect humans and other animals [21]. In a previous study, we showed that MRVs are quite frequently detected in bats and appear genetically more differentiated in comparison with the orthoreoviruses found in other animal species [22]. However, to date, the unique MRVs identified in bats belong to serotypes 2 (Asia) and 3 (Europe) [16,17,23].

This study reports the first isolation and characterization of a new reassortant MRV strain, BatMRV1-IT2011, belonging to serotype 1 from the lesser horseshoe bat (*Rhinolophus hipposideros*) in Italy.

## 2. Results

### 2.1. Sampling

In total, 15 fecal samples were collected from 2009–2015 from the reproductive colony of *R. hipposideros*. During the sampling activities, no relevant mortality or clinical signs indicative of infectious diseases of bats were recorded, and a normal reproductive success rate (number and mortality rate of juveniles until fledging) was observed.

### 2.2. Virological Tests

**Figure 1:**
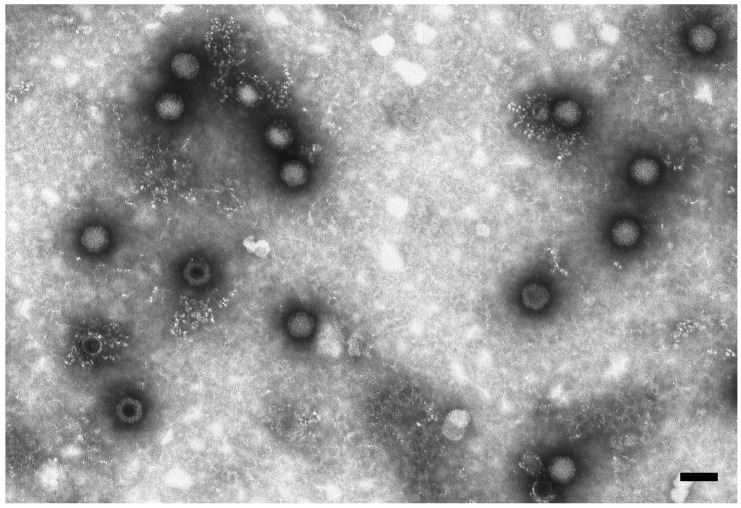
Electron micrograph of reovirus particles in the supernatant of VERO cells. Negative staining (2% sodium phosphotungstate). TEM FEI Tecnai G2 Spirit, 85 kV. Bar = 100 nm.

BatMRV1-IT2011 was isolated by cell culture (Fetal monkey kidney MARC-145) from Sample 191,797 collected in August 2011. The isolate caused a clear cytopathic effects (CPE) at 4–5 days post-inoculation with granulating inclusions, shrinking and falling off. nsEM performed on the supernatant of the infected cell culture revealed a non-enveloped icosahedral virus ~75 nm in diameter, which was morphologically related to the reoviruses (Figure 1). Virus identification was first confirmed by RT-PCR specific for the MRV *L1* gene.

### 2.3. Genome Characterization of BatMRV1-IT2011

The full genome sequence of BatMRV1-IT2011 was determined starting from the cell culture supernatants with CPEs, as previously described [15]. The nucleotide sequences of all 10 genome segments *L1*-*L3*, *M1*-*M3* and *S1*-*S4* were deposited in GenBank under Accession Nos. KT900695–KT900704.

A molecular analysis revealed that BatMRV1-IT2011 was a novel serotype 1 MRV, with an *S1* segment similar to the bovine MRV T1/bovine/Maryland/Clone23/59 and C/bovine/Indiana/MRV00304/2014, but the other segments were more similar to MRVs of different hosts, origins and serotypes. The results of an analysis using the BLAST algorithm showing the highest similarities for each genome segment are reported in Table 1.

The results showed that BatMRV1-IT2011 contains MRV genes that are highly similar to those in viruses detected in bats, humans, cows, civets and pigs, with no date or geographical correlation. Most of the MRVs related to BaMRV1-IT2011 are associated with enteric/respiratory diseases and encephalitis in animals and humans.

**Figure 2:**
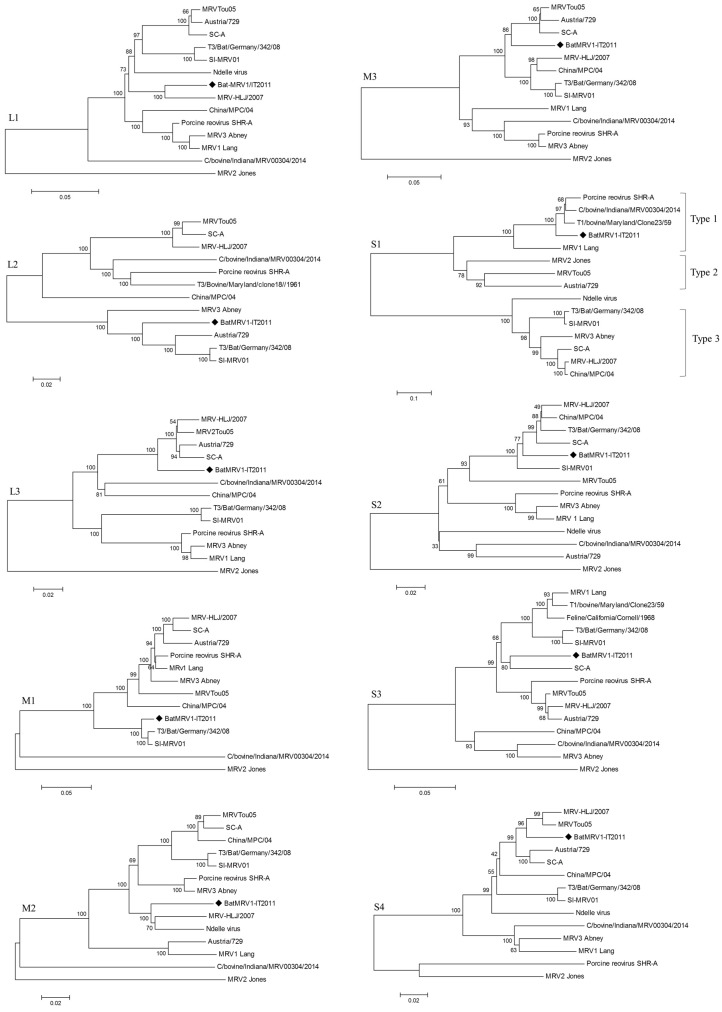
Phylogenetic trees of the BatMRV1-IT2011 strain’s genome segments (♦) and the closest related whole-genome MRV strains from GenBank.

The phylogenetic trees, generated by the neighbor-joining method, for each genome segment are reported in Figure 2.

A phylogenetic analysis revealed discrepancies in the clustering of the 10 genes between BatMRV1-IT2011 and the other MRV strains included in the analysis (Figure 2). In particular, BatMRV1-IT2011 clustered with MRVTou05 based on the *M3* and *S2* segments, but not for the *S1* genomic region, where it was more similar to T1/bovine/Maryland/Clone23/59 and C/bovine/Indiana/MRV00304/2014. This reflects a pattern of topological incongruence caused by reassortment. Unfortunately, the whole genome of T1/bovine/Maryland/Clone23/59 was not available, and for this reason, the C/bovine/Indiana/MRV00304/2014 strain was included in the Recombination Detection Program RDP analysis, even though it was isolated after the putative reassortant strain described here.

**Table 1:** Highest nucleotide identities for each gene segment of the novel BatMRV1-IT2011. MRV, mammalian orthoreovirus.

BatMRV1-IT2011	Similarity (%)	MRV Strain	Serotype	Host	Disease	Country	GenBank Accession No.	Encoding Protein and Function
***L1***	93	MRV-HLJ/2007	3	Pig	Fever, respiratory illness	China	HQ642769.1	λ3—RNA-dependent RNA polymerase
	90	Porcine reovirus SHR-A	1	Pig	NA	China	JX415466.1	
***L2***	91	Austria/729	2	Pig	Encephalitis	Austria	JN799427.1	λ2—Guanylyltransferase, methyltransferase
	90	T3/bat/Germany/342/08	3	Bat	Hemorrhagic enteritis	Germany	JQ412756.1	
	90	SI-MRV01	3	Human	Acute gastroenteritis	Slovenia	KF154725.1	
***L3***	95	MRVTou05	2	Human	Encephalitis	France	GU196308.1	λ1—Helicase, binds dsRNA, NTPase
	94	MRV-HLJ/2007	3	Pig	Fever, respiratory illness	China	HQ642769.1	
***M1***	98	T3/bat/Germany/342/08	3	Bat	Hemorrhagic enteritis	Germany	JQ412758.1	μ2—NTPase
	98	SI-MRV01	3	Human	Acute gastroenteritis	Slovenia	KF154727.1	
***M2***	92	MRV-HLJ/2007	3	Pig	Fever, respiratory illness	China	HQ642773.1	μ1—Cell penetration, apoptosis
	92	4 Ndelle virus	Putative 4	Mouse	NA	Cameroon	AF368034.1	
***M3***	93	MRVTou05	2	Human	Encephalitis	France	GU196314.1	μNS—Nucleates viral inclusion bodies
	92	Austria/729	2	Pig	Encephalitis	Austria	JN799425.1	
***S1***	90	T1/bovine/Maryland/Clone23/59	1	Bovine	NA	USA	AY862134.1	σ1, σ1s—Viral attachment
	88	C/bovine/Indiana/MRV00304/2014	1	Bovine	Diarrhea	USA	KJ676385.1	
***S2***	95	China/MPC/04	3	Civet	NA	China	GQ468273.1	σ2—Inner capsid structural protein
	94	T3/bat/Germany/342/08	3	Bat	Hemorrhagic enteritis	Germany	JQ412762.1	
	94	SI-MRV01	3	Human	Acute gastroenteritis	Slovenia	KF154731.1	
	94	MRV-HLJ/2007	3	Pig	Fever, respiratory illness	China	HQ642776.1	
***S3***	91	SC-A	3	Pig	Diarrhea	China	DQ411553.1	σ2—ssRNA-binding
	91	Feline/California/Cornell/1968	3	Cat	NA	USA	U35362	
***S4***	95	MRVTou05	2	Human	Encephalitis	France	GU196313.1	σ3—DS-RNA binding, modulation of cellular interferon response
	95	MRV-HLJ/2007	2	Pig	Fever, respiratory illness	China	HQ642778.1	

Notes: L, large segments; M, medium segments; S, small segments; NA, not available.

**Figure 3:**
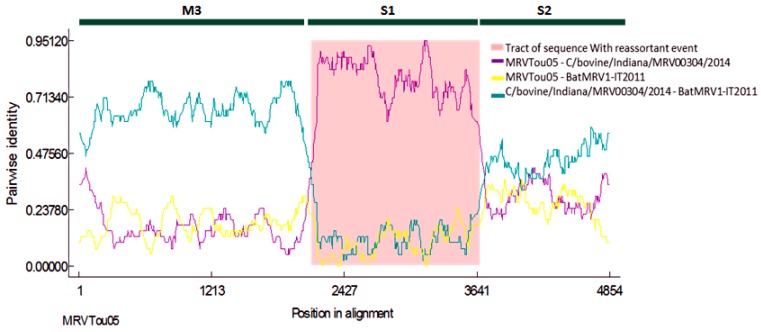
Similarity plots of BatMRV1-IT2011. M3, S1 and S2 are the three reovirus viral genes on which the RDP analysis was performed. Recombinant: IT2011; major parent: MRVTou05; minor parent: MRV00304-2014. The specific algorithms used were RDP, GENECONV, Chimaera, MaxChi, BootScan and SiScan implemented in the RDP4 software

An RDP analysis was performed on three genes (*M3*, *S1*, *S2*) to better describe the reassortant event. In Figure 3, it is shown that BatMRV1-IT2011 was derived from a cross-over event, which probably occurred between strains that had sequences that were highly related to MRVTou05 and C/bovine/Indiana/MRV00304/2014. Indeed, RDP4 detected well-supported breakpoints near the boundaries of the S1 segments in the concatenated genomes, suggesting that the phylogenetic incongruence was significant among the three regions. All of the algorithms indicated that the newly-reassortant strains had arisen by acquiring the *S1* genome segment from a strain belonging to serotype 1 in which the *S1* gene was closely related to C/bovine/Indiana/MRV00304/2014.

To better investigate the differences in BatMRV1-IT2011, we aligned the deduced amino acid (aa) sequence of the σ1 protein with those from the strains T1/bovine/Maryland/Clone23/59, C/bovine/Indiana/MRV00304/2014, porcine reovirus SHR-A and MRV type 1 Lang. The aa sequence comparison revealed a 90% identity between BatMRV1-IT2011 and the bovine strains with differences of 49 and 44 aa for C/bovine/Indiana/MRV00304/2014 and T1/bovine/Maryland/Clone23/59, respectively (Figure S1). The aa sequence comparison between BatMRV1-IT2011 and porcine reovirus SHR-A and MRV type 1 Lang revealed an 85.6% and 79.0% identity, respectively. Phylogenetic analysis of the deduced aa sequence from BatMRV1-IT2011 is reported (Figure S1).

### 2.4. VNTs for Virus Typing and Serology

VNTs confirmed that BatMRV1-IT2011 belongs to serotype 1, being strongly neutralized at a high titer (1/1280) from a guinea pig immune serum produced against the MRV T1L and no or low neutralization by anti-T2J and anti-T3A immune sera, respectively (Table 2).

Additionally, a high neutralizing antibody rate was found in the majority of serum samples from different mammalian species (cows, pigs, horses and dogs) against the MRVs T1L, T2J, T3A and BatMRV1-IT2011. VNTs also showed reciprocal cross-reactions between BatMRV1-IT2011 and the reference strain T1L in serum samples of all of the mammalian species (Figure S2).

**Table 2:** MRV neutralizing antibody titers of rabbit and guinea pig sera produced against the MRV reference strains type 1 Lange (T1L), type 2 Jones (T2J) and type 3 Abney (T3A).

Immune Serum	Virus (100 TCID_50_/25 µL)			
	MRV1 Lang	MRV2 Jones	MRV3 Abney	BatMRV1-IT2011
MRV negative (rabbit)	0	0	0	0
MRV negative (guinea pig)	0	0	5	0
MRV1 Lang (guinea pig)	1280	80	40	1280
MRV2 Jones (rabbit)	5	80	10	20
MRV3 Abney (guinea pig)	0	0	320	20

Results are expressed as the reciprocal of the final serum dilution required to neutralize 100% of the inoculated cultures with a viral concentration of 100 tissue culture infectious dose 50% (TCID_50_)/25 µL.

## 3. Discussion

In our previous study, a virological survey on bat populations in Italy, we characterized 15 MRVs and determined that MRV type 3 is the most widespread among bats [16]. A similar study conducted in Germany confirmed this observation [17]. Recently, a reassortant MRV belonging to serotype 2 has been detected in bats in China [23]. Therefore, to the best of our knowledge, before this report, the only MRVs identified in bats belonged to serotypes 2 and 3. Here, we report the isolation and characterization of a new serotype 1 MRV from the lesser horseshoe bat (*R. hipposideros*) named BatMRV1-IT2011, with evidence of an *S1* genome segment reassortment.

In addition, new original data and information on the dynamics and epidemiology of MRV infections in bats were obtained through the observation and sampling of a reproductive bat colony from May–September over a six-year (2009–2015) period. Only one sample, collected in August 2011, out of 15 total fecal samples collected during the entire study was positive for MRV. Thus, we demonstrated that the MRV infection in the lesser horseshoe bat is likely transitory.

Since the sole reassortant bat MRV was previously described in *Rhinolophus pusillus* and we detected a reassortant MRV in bats of the same genus, this could indicate that members of the genus *Rhinolophus* play a key role in the generation of new MRVs characterized by genome segment reassortments and unpredictable biological properties. The source of the MRV infection in bats has not been determined, nor has the origin of the genomically-different viruses. The transmission of reoviruses from one host to another is not limited to close contacts, but can occur due to indirect contamination. Infection through contaminated food, water or other factors in the environment is highly possible, since infective reovirus particles have been found in environmental samples [24,25,26,27]. Viral persistence outside the host is an advantageous feature that enables them to spread efficiently. As an additional possibility, MRVs could be mechanically transmitted to insectivorous bats and other mammals through insect vectors, although there is not yet evidence for this type of transmission [18].

The absence of mortality or clinical signs indicative of infectious diseases in the bat colony during the six-year observation period is not surprising, since the order *Chiroptera* is the most resistant of all mammals to viral infection. However, bats are known to be reservoirs and the sources of a variety of viruses with zoonotic potential.

This study also investigated the presence of neutralizing antibodies against BatMRV1-IT2011, as well as T1L, T2J and T3A in animals. The preliminary results suggested that MRVs are widespread in animals and that infections containing multiple serotypes, including MRVs of serotype 1 with a *S1* gene similar to BatMRV1-IT2011, are common.

Similar trends in reovirus infections containing multiple serotypes have been previously reported in healthy dogs [28,29,30], cattle [31], swine [32] and humans [9,33,34]. Infections with multiple MRVs represent the basis for the genesis of new reassortant strains in permissive hosts, and insectivorous bats may play an important role in this genesis. For a better understanding and knowledge of the genetic diversity, origin and mechanisms involved in the genetic exchange of MRVs, researchers will need to obtain and study the whole-genome sequences of a large number of isolates collected from different hosts and countries.

The cooperation among veterinary virologists, bat experts and public health bodies with highly specialized diagnostic laboratories is an essential point for collaboration on topics concerning both animal and human health that are aimed to establish an innovative and suitable intervention in the case of emerging infections.

In conclusion, our results extend the current knowledge on bat MRVs and stress the importance of continued and improved *Orthoreovirus* surveillance in bats and other mammals, along with the development and standardization of specific diagnostic tools.

## 4. Materials and Methods

### 4.1. Sampling

**Figure 4:**
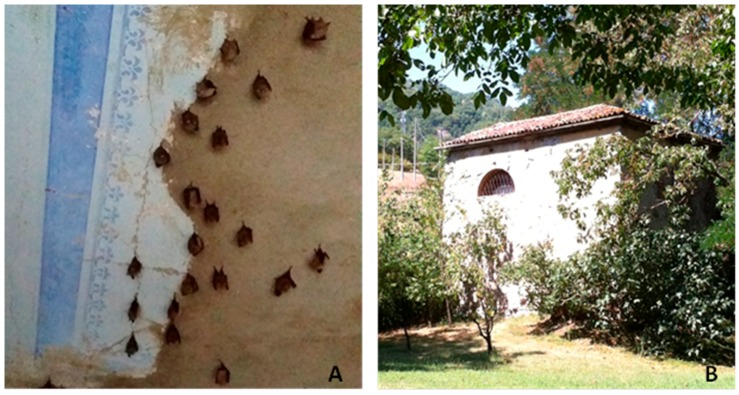
(**A**) Reproductive colony of lesser horseshoe bats (*Rhinolophus hipposideros*) sampled in the study and (**B**) its building roost.

Fresh fecal samples were collected for virological investigations from a known and georeferenced reproductive colony (44°17′46,36′′N, 11°7′3,79′′E) of lesser horseshoe bat (*Rhinolophus hipposideros*), which roosts in an old chapel located in the Vergato Province of Bologna, northern Italy (Figure 4). The bat species was identified based on morphologic characteristics according to European bat identification keys [9]. Samples were collected during the period of bat activity from 2009–2015 using a clean plastic sheet placed under the roost site for ~24 h before sampling. The survey did not encompass any direct manipulations of the bats and relied entirely on the collection of fecal samples, which were submitted to the laboratory and stored at −80 °C until processing.

### 4.2. Viral Isolation

Fecal samples were homogenized in minimal essential medium (1 g/10 mL) containing antibiotics and clarified by centrifugation at 3000× *g* for 15 min. Samples were inoculated in confluent monolayers of VERO (African green monkey) and MARC-145 (Fetal monkey) kidney cells, incubated at 37 °C with 5% CO_2_ and observed daily for 7 d to highlight the development of (CPEs). In the absence of CPEs, the cryolysates were sub-cultured twice onto fresh monolayers.

### 4.3. Electron Microscopy

Supernatant fluids from cell cultures showing CPEs were submitted for negative-staining electron microscopy (nsEM) using the Airfuge method [35]. Grids were stained with 2% NaPT, pH 6.8, for 1.5 min and examined at 19,000–30,000× using a Tecnai G2 Spirit transmission electron microscope (FEI, Eindhoven, NL, The Netherlands) operating at 85 kV and equipped with an Olympus Veleta digital camera. Viral particles were identified based on their morphological characteristics.

### 4.4. Molecular Testing and Analysis

RT-PCR with specific primers for a conserved region of the *L1* viral gene, which is common to different MRV serotypes, was used for viral identification [8]. To obtain the whole genome sequence of the isolate, the Ion Torrent next-generation sequencing platform was used as previously described [15]. Searches using the BLAST algorithm were performed on the NCBI server [36] with the available database. The sequence alignments of the 10 gene segments (*L1*–*L3*, *M1*–*M3* and *S1*–*S4*) were compared with those of reference strains and other orthoreoviruses downloaded from GenBank using the program ClustalW. Phylogenetic trees for each genome segment were generated using the neighbor-joining method with the Kimura 2-parameter model. Branch support was assessed by the bootstrap analysis of 1000 replicates. Putative reassortant viruses were preliminarily identified by their topological incongruities among all of the phylogenies. The reassortant viruses were further investigated using a small set of the full genome sequences of the closest related MRVs available from GenBank. Only strains for which the complete genome was available in GenBank were included. The 10 gene segment alignments were concatenated in the order of their length to generate a single alignment of complete genome sequences, which was further analyzed using a recombination detection program, RDP4. The specific algorithms used were RDP, GENECONV, Chimaera, MaxChi, BootScan and SiScan implemented in the RDP4 software using default settings [37]. We used more than one method to analyze the data, because evaluations of these recombination detection methods, using both simulated and empirical data, have shown that the results from only a single method are not very reliable [38]. The hypothesis of reassortment was supported when the recombinant breakpoints were detected near the junctions when the genome segments were manually concatenated.

### 4.5. Virus Neutralization Test

The Virus neutralization test (VNT) was carried out in 96-well microplates using BatMRV1-IT2011 grown on MARC-145 cells, and rabbit and guinea pig immune sera produced against the MRV prototype strains, type 1 Lang (T1L), type 2 Jones (T2J) and type 3 Abney (T3A).

Serial two-fold dilutions of each immune serum (2 wells/dilution) in 25 μL of serum-free culture medium were added to each well and incubated for 1 h at 37 °C with an equal volume of tissue culture fluid containing 100 tissue culture infectious dose 50% (TCID_50_) of BatMRV1-IT2011. Virus back titrations of the working virus dilution were included, using six wells per 10-fold dilution, to confirm the validity of the test results. A 50 μL volume of cells at log5 cells/mL in medium containing 10% fetal calf serum was added to each well. After incubation for 5–6 days at 37 °C with 5% CO_2_, wells were scored for CPEs, and neutralizing titers were expressed as the reciprocal of the final serum dilution required to neutralize 90% of the inoculated cultures.

For a preliminary verification of the eventual circulation and diffusion of this and other MRVs in other animals, we tested 50 serum samples collected from different species (20 cows, 10 pigs, 10 horses and 10 dogs) for the presence of neutralizing antibodies against BatMRV1-IT2011 and T1L, T2J or T3A. The serum samples were randomly collected from animals residing within 200 km of the site of the bat roost.

## Supplementary Materials

Supplementary materials can be found at http://www.mdpi.com/1999-4915/7/11/2908/s1.
