# Supplementary material for: Detection and Characterization of a Novel Reassortant Mammalian Orthoreovirus in Bats in Europe
Source: Viruses. 2015 Nov 11;7(11):5844–54. doi: 10.3390/v7112908 (PMC4664981; doi:10.3390/v7112908)
Supplement: Supplementary file 1 [file viruses-07-02908-s001.pdf]

viruses

MDPI

Supplementary materials

```
!Domain=Data;
#BatMRV1-IT2011                    MDASLISEIR RIVLQLTQSN NGSIDKELKE IKKQVEINVT DIRNTNLRLD GIRGQVDGIS DSVAAIERRL GEVDGRLVAI  [ 80]
#C/bovine/Indiana/MRV00304/2014    .....T.... .......... .......... ..E......N ......IK.. ......T... ..I....... ........V.  [ 80]
#T1/bovine/Maryland/Clone23/59     .....T.... .......... ....N..F.. ..E....... .......K.. ......AS.. .......... ........V.  [ 80]
#Porcine_reovirus_SHR-A            .....T.... .T.......S ....N..... ..E..D...A .......K.. ...R..T... .......... ....D...V.  [ 80]
#MRV1_Lang                         ......T... K.....SV.S ...QS..IE. .....QV..D ...AA.IK.. .LGR.IAD.. N.IST..S.. ..M.N...G.  [ 80]
#MRV2_Jones                        .SDLVQLIR. E.L.LTGNGE SANSKH.IE. ....IKDISA .VNRISNIV. S.Q..LG.L. VR.S...SGV S.NGN.IDRL  [ 80]
#MRV3_Abney                        ..PR.RE.VV .LIIA..SD. GI.LS.G.ES RVSAL.KTSQ IHSD.I..IT QGLDDANKRI IALEQSRDD. VASVSDAQLA  [ 80]

#BatMRV1-IT2011                    TNQLTQLSNS VGQNTQDISS MGNRLDAIEP RVDDLDTLTY NLAGRTSTLE ADVGGIRTEL SALTTRVTTE VARLDALISA  [160]
#C/bovine/Indiana/MRV00304/2014    ...I...... .S.......A ..D..N.V.. .I.......S .......... T...V.A... .......... .........D  [160]
#T1/bovine/Maryland/Clone23/59     ...I...... .S.......A ..D..N.... .I.......S .......... T.....A... ......I... ..........  [160]
#Porcine_reovirus_SHR-A            ...I...... .S.......A ..D..N.V.. .IN......S D.VR...... T.....A... .......... .........V  [160]
#MRV1_Lang                         SS.V...... .S....S... L.D.IN.V.. ...S...V.S ..T....... ....SL.... A......... .T...G..NS  [160]
#MRV2_Jones                        ERDVSGI.A. .SGIDSRL.E L.D.VNVA.Q .IGQ...V.D ..LE.A.R.. TE.SA.TND. GS.N...... LNDVRQT.A.  [160]
#MRV3_Abney                        ISR.ESSTGA LQTVVNGLD. SVTQ.G.RVG QLETGLAELR VDHDNLVARV DTAERNIGS. TTELSTL.LR .TSIQ.DFES  [160]

#BatMRV1-IT2011                    GQTSLTGLSS RLDAAEAAMV TTAGQGLRKD GNTLNVIVGN GMWFNNSNQL QLDLSGQAKG VGFEGSGMVA KIDTNYFSYN  [240]
#C/bovine/Indiana/MRV00304/2014    ..S...S... ....V..V.I .......... .......... .......... .....N.... .......V.V ..........  [240]
#T1/bovine/Maryland/Clone23/59     ..A...S... ....V..V.I .......... .......... .......... .....N.... .....G.V.. ..........  [240]
#Porcine_reovirus_SHR-A            ..S...SV.. ....V..V.I .......W.. ......NI.. ..R....... .....N.... I....N.... ..........  [240]
#MRV1_Lang                         ..N.IGE..T ..SNV.TS.. ....R..Q.N .......... .....S.... .......S.. ...V.T...V .......A..  [240]
#MRV2_Jones                        ID.R..T.ET DAVTSVGQGL QKT.NSIKVI VG.GMWFDR. NVLQLFVSNQ .KG.GFIDN. MVVKIDTQYF SF.S.GNITL  [240]
#MRV3_Abney                        RIST.ERTAV TSAG.PLSIR NNRMTMGLN. .L..SGNNLA IRLPG.TGLN IQNGGL.FRF NTDQFQIVNN NLTLKTTVFD  [240]

#BatMRV1-IT2011                    SNGEITLVSQ INGLPARVSS LESLKIDTIL PPLTVREASG IRTLSLGYDT SDFTIINSVL ALRSRLTLPT YRYPLELDAS  [320]
#C/bovine/Indiana/MRV00304/2014    ........N. .........T .......... ....LS.... A.......E. N.......M. S......... ........T.  [320]
#T1/bovine/Maryland/Clone23/59     ........N. .S.......T .......... .....S.... A......... N......... S......... ..........  [320]
#Porcine_reovirus_SHR-A            ........N. .........T .......... .....S.V.. A.......EI N...T..... S..AK..... ..........  [320]
#MRV1_Lang                         .......... ..E..S...T ...A...SV. .......... V...F..... .......... S......... ........TA  [320]
#MRV2_Jones                        N.NISG.PAR TGS.E.SRID VVAPPLVIQS TGS.RLLRLM YEAVDFVVTN NVL.LR.RSV TPTFKFP.EL NSADNSVSIH  [320]
#MRV3_Abney                        .INSRIGATE QSYVASA.TP .RLNSSTKV. DM.IDSSTLE .NSSGQLTVR .TSPNLRYPI .DV.GGIGMS PN.RFRQSMW  [320]

#BatMRV1-IT2011                    NNRVQIAERF GMRTGSWTGQ LQYQHPQLSW QTSVTVNLMK VDDWLVLSFA QMTTNGIMAD GKFVINFVSG LSSGWQTGET  [400]
#C/bovine/Indiana/MRV00304/2014    .......... .......... .......... .AN....... .........T ....G..... ...T...L.. ..........  [400]
#T1/bovine/Maryland/Clone23/59     .......... .......... .......... .AN....... .........T ....S..... ...I...L.. ..........  [400]
#Porcine_reovirus_SHR-A            .....M.... .......... ........N. .AN....... .........T ....G..... ...T...S.. ..........  [400]
#MRV1_Lang                         .....V.D.. .....T.... .......... RAN..L.... .........S .....S.... .......... ........D.  [400]
#MRV2_Jones                        R.YRIRLGQW SGQLEYH.PS .RWNA.VTVN LMR.DDW.IL SFTRFST.GI LASGKFVLNF VTGLSPGWAT G.TEPS.TTN  [400]
#MRV3_Abney                        IGI.SYSGSG LNWRVQVNSD IFIVDDYIHI CLPAFNGFSI A.GGDLSLNF VTGLLPPLLT .DTEPA.HND VVTYGAQTVA  [400]

#BatMRV1-IT2011                    QPSSTTDPLS TTFAAVQFLN ASGNRVDAFR IMGVSEWTDG ELEIKNYGGT YTGHTQVYWA PWTIMYPCNV R* [472]
#C/bovine/Indiana/MRV00304/2014    .......... ...V...Y.. ...T...... .......V.. .......... ..S....... .......... .. [472]
#T1/bovine/Maryland/Clone23/59     .......... ...V...Y.. .G.T...... .......I.. .......... ..S....... .......... .. [472]
#Porcine_reovirus_SHR-A            .......... .A.V...Y.K ...T...... ...I...V.. .......... .......... .......... .. [472]
#MRV1_Lang                         E....I..W. .......... N-.Q.I.... .......... .......... .......... .......... .. [472]
#MRV2_Jones                        PL.T.FAAIQ FINGSSRVDA FRILG.AEWN AGELEITNY. GTYTAHTNVD WAPM.IM.PC LG*------- -- [472]
#MRV3_Abney                        IGL.SGGAPQ YMSKNLWVEQ WQDGVLRLRV EG.G.ITHSN SKWPAMTVSY PRSF.*---- ---------- -- [472]
```

Figure S1. Deduced amino acid sequences of the σ1 protein of BatMRV1-IT2011 and the strains T1/bovine/Maryland/Clone23/59, C/bovine/Indiana/MRV00304/2014T3D/55, Porcine_reovirus_SHR-A, MRV type 1 Lang, MRV type 2 Jones and MRV type 3 Abney.

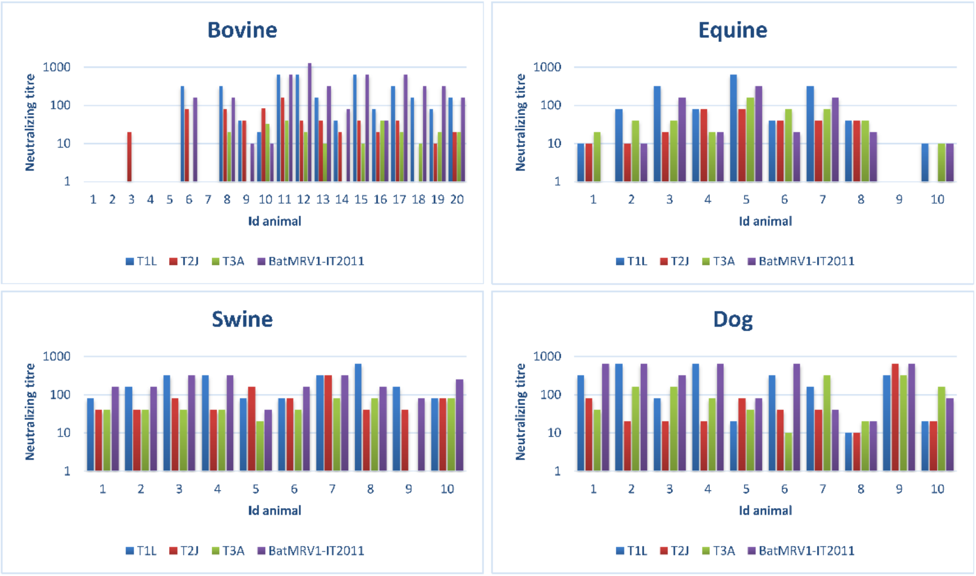

Figure S2. Virusneutralization: presence of neutralizing antibodies against BatMRV1-IT2011, MRV Type 1 Lang, Type 2 Jones and Type 3 Abney in serum samples of different species.
